# Development and Characterization of the Shale Stratum Well Wall Stabilized with Nanosomal Sealing Agent

**DOI:** 10.3390/polym16121614

**Published:** 2024-06-07

**Authors:** Daqi Li, Shuyang Gao, Zhichuan Tang, Huimei Wu, Yayun Zhang

**Affiliations:** 1 State Key Laboratory of Shale Oil and Gas Enrichment Mechanisms and Effective Development, Beijing 102206, China; lidq.sripe@sinopec.com (D.L.); gaosy.sripe@sinopec.com (S.G.); tzc27889.sripe@sinopec.com (Z.T.); zhangyy.sripe@sinopec.com (Y.Z.); 2 Sinopec Research Institute of Petroleum Engineering Co., Ltd., Beijing 102206, China; 3 School of Petroleum Engineering, Yangtze University, Wuhan 430100, China

**Keywords:** polymer, temperature-sensitive, sealing agent, borehole stability

## Abstract

The development of micro cracks in shale formations can easily lead to wellbore instability caused by liquid phase invasion. In order to effectively seal the shale micropores, the surface treatment of nano-SiO_2_ particles was developed using the silicane coupling agent A-1891. Then, the temperature-sensitive polypenic acrylamide polymer was modified onto the surface of the nanoprocal particle through reaction to obtain the nanosomal blocking agent ASN. The infrared spectrum shows that there are chemical bonds between the generated polymer chains, rather than simple physical composites, indicating the successful synthesis of the temperature-responsive nanosealing agent ASN. The particle size analysis showed that the synthesized nanoparticles in ASN have a uniform particle size distribution and display no agglomeration phenomenon. Applying ASN as a sealing agent in drilling fluid effectively fills the nanoscale micropores and microcracks in shale, making shale denser and significantly improving the wellbore stability of shale formations. In addition, it has good temperature resistance, can adapt to reservoirs at different temperatures, is non-toxic and environmentally friendly, and has good prospects for stable applications in shale formation wellbore.

## 1. Introduction

Due to the highly developed, weak surface structures, such as the nanoscale microcracks and bedding planes in shale formations, as well as their strong water sensitivity, serious wellbore stability problems are prone to occur during long horizontal drilling [1,2,3,4]. In shale formations, their inherent porosity and permeability are both at extremely low levels. Consequently, a minimal intrusion of filtrate will result in an increase in pore pressure near the wellbore. This rise in pressure severely weakens the effective mechanical support that the fluid column originally provided for the wellbore wall, thereby heightening the risk of wellbore collapse and instability [5,6,7]. Maintaining the stability of the wellbore crucially hinges on effectively containing the transmission of pore pressure [8,9]. To effectively prevent the transmission of pore pressure, it is necessary to strengthen the reinforcement sealing measures for the pores [10]. Mud shale has extremely low permeability (around 1 × 10^−9^ µm^2^), coupled with minuscule pore throat dimensions. Conventional sealing agents, such as asphalt-based and polymeric alcohol-based materials [11], are often employed for wellbore stabilization and collapse prevention. However, their relatively large particle sizes (micron-scale) pose challenges in penetrating the nanometer-scale pore and fracture structures of shale, resulting in the inability to form a tight sealing layer. This limitation not only undermines wellbore stability but also hampers the protection of the reservoir [12].

In recent years, researchers have primarily focused their studies on nanoscale sealing agents for drilling fluids, particularly in the realm of inorganic nanoparticles, organic nanoparticles, and inorganic/organic composite nanoparticles. Inorganic nanoparticles exhibit remarkable properties like superior surface energy, rigidity, impeccable dimensional stability, and excellent thermal stability and are exemplified in materials such as graphite [13], silica [14], calcium carbonate [15], etc. However, their large specific surface area makes them prone to agglomeration and poor dispersion, significantly affecting the sealing performance [16]. Organic nanoparticles have strong elasticity and can be compressed and deformed under certain temperatures and pressures to enter the shale formation for plugging. They are mainly nanopolymer microspheres or a nanolotion synthesized with one or more monomers, such as styrene, acrylic acid, methyl methacrylate, etc. However, the environmental impact of polymers restricts their large-scale application [17]. Inorganic/organic composite nanoparticles are mainly surface-modified with polymers. Through the integration of the rigidity, dimensional stability, and thermal endurance of inorganic materials with the resilience, processability, and nodal efficiency of polymers, we can harness the synergistic and complementary properties of these components to yield polymer nanocomposites of exceptional performance. This area has become a hot topic in research [18,19,20,21].

In addition, complex underground environmental stimuli, especially temperature stimuli, have increased the responsive requirements for nanosealing agents [22,23,24]. This study combines nanomaterial technology, polymers, and drilling fluid technology. The silane-coupling agent A-1891 is selected to treat the surface of the nano-SiO_2_ particles. Double bonds are modified onto the surface of the nanoparticles. Then, temperature-sensitive poly(N-isopropylacrylamide) polymers are modified onto the surface of the nanoparticles to obtain temperature-responsive nanoparticles (ASNs). As the temperature varies, the hydrophilic/hydrophobic properties of the nanoparticle surface undergo corresponding changes. Furthermore, the temperature-sensitive properties of poly(N-isopropylacrylamide) can also be modulated via the introduction of hydrophilic or hydrophobic monomers, which can obtain nanoparticles that undergo a hydrophilicity/hydrophobicity transition at different temperatures. The application of the nanoparticles as sealing agents in drilling fluids can effectively fill the shale. In addition, nanoscale micropores and microcracks make shale denser, significantly improving the wellbore stability of shale formations. The nanoparticles have good temperature resistance, can adapt to reservoirs at different temperatures, are non-toxic and environmentally friendly, and have good prospects for stable application in shale formation wellbores.

## 2. Experimental Section

### 2.1. Experimental Materials

The nano SiO_2_ (particle size 10–20 nm) and N-isopropylacrylamide (NIPAM) were purchased from the National Pharmaceutical Group Chemical Reagents. The silane-coupling agent A-1891 was purchased from Qufu Yishun Chemical Co., Ltd. (Qufu, China), and the acrylic acid (AA), potassium persulfate (KPS), and tetrahydrofuran (THF) were purchased from the Aladdin Chemical Reagent Company (Shanghai, China). The experimental water was deionized water.

### 2.2. Sample Preparation

#### 2.2.1. Surface Modification of Nano SiO_2_

The siloxanes and hydroxyl groups on the surface of SiO_2_ give it significant hydrophilicity, and there are intermolecular forces leading to the strong aggregation of SiO_2_ [3]. Therefore, the biggest challenges faced in the preparation and application processes of polymer/inorganic nanoparticle composites are in ensuring an adequate dispersion of inorganic nanoparticles in the polymer matrix and strengthening the interaction between the polymer matrix and inorganic nanoparticles [25]. Therefore, the physical or chemical modification of the SiO_2_ surface is the key to preparing uniformly dispersed polymer composite materials. In addition, the modification of the silane-coupling agent is the most commonly used and traditional modification method [26,27]. The chemical bond theory [28] suggests that organic silane-coupling agents first undergo hydrolysis under the action of moisture in the air, and then dehydrate and condense to form oligomers. These oligomers subsequently establish hydrogen bonds with the hydroxyl groups which are present on the silica surface and undergo a dehydration reaction through heating and drying to produce partial covalent bonds, thereby covering the surface of the silica with silane-coupling agents.

The nano-SiO_2_ particles were subjected to a thorough drying process under vacuum conditions at 105 °C for a period of one day to eliminate any residual moisture. Then, we weighed a certain amount of nano SiO_2_ and dispersed it in 50 mL anhydrous ethanol, and sonicated it at a certain power for 1 h. We dissolved 6 g of silane-coupling agent A-1891 in 10 mL of anhydrous ethanol and mixed it well [29,30,31]. Then, we continued the ultrasound treatment for 1.5 h and transferred it to a four-necked flask and reacted it for 6 h under N_2_ atmosphere, uniform stirring, and ultrasonic oscillation at 95 °C. After the reaction was complete, we centrifuged the product and washed it several times with anhydrous ethanol, before drying the sample in a vacuum environment at 65 °C and labeling it as A-1891-nano-SiO_2_ for later use.

#### 2.2.2. Synthesis of ASN Nanosealing Agent

**Figure 1:**
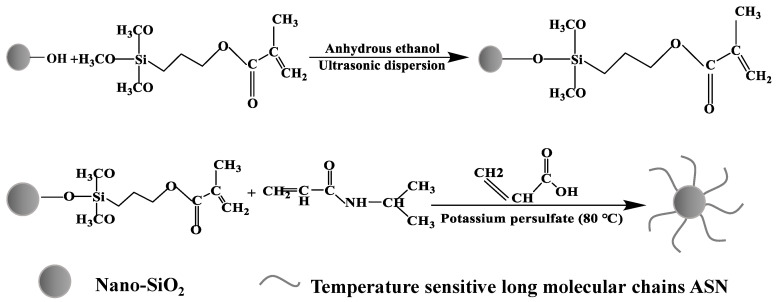
The synthesis process of the temperature-responsive nanosealing agent ASN.

NIPAM and AA were dissolved in a 2:1 volume ratio H_2_O/THF mixed solvent, maintaining a specific molar ratio between the two components. An appropriate amount of A-1891-nano-SiO_2_ nanoparticles was added; these were sonicated for 30 min, a certain amount of potassium persulfate was added, and the mixture was quickly stirred to make the system uniform. The reaction was performed at 85 °C under N_2_ protection for 9 h (Figure 1). After the reaction, the product was centrifuged, washed several times with anhydrous ethanol, dried in a 65 °C vacuum drying oven, and marked as ASN for future use.

### 2.3. Temperature-Responsive Nanosealing Agent Characterization

The sample underwent rigorous testing with the Bruker Optics Tensor 37 instrument for accurate analysis, employing the KBr compression method, with a resolution of 4 cm^−1^ and a scanning range of 4000–400 cm^−1^. The STA 449F3 TGA/DSC synchronous thermal analyzer (Netzsch, Germany) was used for the sample thermogravimetric TG-DTA analysis, in a N_2_ atmosphere, and a heating range of 30–1500 °C. Using the MesoMR23-060V-I nuclear magnetic resonance core analyzer (Shanghai Newmai Technology Co., Ltd., Shanghai, China), the pore size distribution characteristics before and after shale sealing were quantitatively characterized to detect the mobile phase in the rock. The shale was not damaged; therefore, the pore size distribution, porosity, permeability, etc., of the rock sample could be analyzed. There are no special requirements for the shape of the core, and it can be tested repeatedly [32,33,34,35]. The sample was subjected to particle size testing using the Marvin Panaco NanoSight NS300 (Malvern, Britain) laser particle size analyzer.

### 2.4. Comprehensive Performance Evaluation

#### 2.4.1. Core Sealing Performance

**Figure 2:**
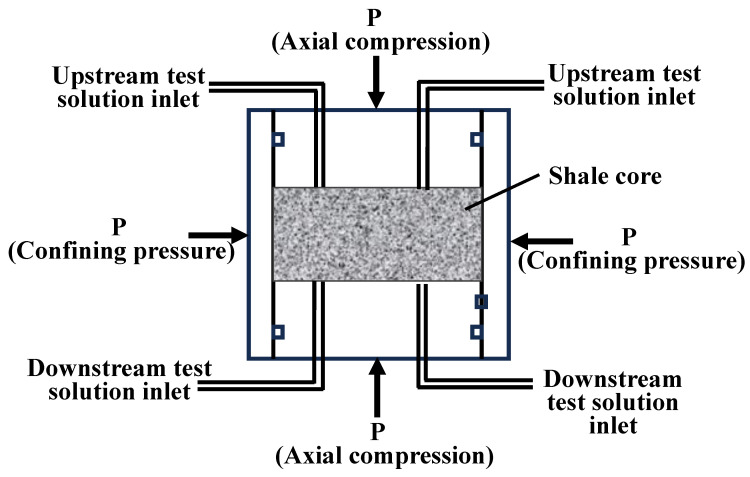
A schematic diagram of the shale pressure transmission technology.

The permeability of shale is extremely low (10^−6^–10^−12^ μm^2^); therefore, Darcy’s law is not applicable. However, pressure transfer methods can be used for detection [36,37]. Shale pressure transfer technology provides a scientific and effective experimental means for optimizing and evaluating the performance of anti-collapse drilling fluid systems and is one of the principal indicators of the important progress in the mud shale wellbore stability research in recent years [38]. Through the development of a shale hydration–mechanical coupling simulation experimental device and the establishment of a pressure transmission evaluation method, we aimed to assess the performance of sealing agents in mitigating shale pressure transmission and resisting filtrate invasion. At the same time, we also precisely measured the key parameters relating to shale permeability to ensure the accuracy and reliability of the experimental results. The schematic diagram of the pneumatic transmission technology of the instrument is shown in Figure 2. The shale core is securely positioned within the core gripper, subsequently pumping the test fluid precisely through the upstream inlet to guarantee thorough and even contact across the entire upper end face of the core, maintaining a pressure of 2.1 MPa at the core tip and an initial pressure of 1.0 MPa at the bottom. Through the monitoring of changes in the bottom pressure, the pressure transfer rate of the test solution (Formula (1)) is obtained to evaluate its sealing performance.

(1) $$K=\left(\frac{\mu \beta VL}{A}\right)\times \left(\ln\left(\frac{P_{m}-P_{0}}{P_{m}-P\left(L,t_{2}\right)}\right)-\frac{\ln\left(\frac{P_{m}-P_{0}}{P_{m}-P\left(L,t_{1}\right)}\right)}{\left(t_{2}-t_{1}\right)}\right)$$

In Formula (1), “*K*” is the notation for shale permeability, and its measurement unit is μm^2^. “*μ*” is used to describe the viscosity properties of a fluid, with its unit being MPa·s. “*β*” is a parameter that quantifies the static compressibility of a fluid, with its unit being MPa^−1^. “*V*” represents the volume of the downstream container, specifically in units of cm^3^. “*L*” refers to the length, with its unit being cm. “*A*” identifies the cross-sectional area, specifically in units of cm^2^. “*T*” is the representation of the time required for pressure transmission, with its unit being seconds (s). “*P_m_*” stands for the pressure value upstream of the core sample, measured in MPa. “*P*_0_” identifies the initial pressure within the pores of the core sample, also measured in MPa. “*P*(*L*,*t*)” is a function that describes the downstream pressure value at a distance *L* from the core inlet after a time t, with its unit also being MPa.

#### 2.4.2. Temperature Response Performance

For temperature-sensitive polymers, conducting permeability measurements across multiple temperatures offers a swift and reliable method for validating their temperature responsiveness [39,40]. When compared to the lower critical solution temperature (LCST) threshold, the external temperature is found to be lower and the temperature-sensitive polymer exhibits hydrophilic properties, leading to a high light transmittance.

#### 2.4.3. Wetting Performance

Through core wettability experiments, the wettability characteristics of rocks can be understood. The experiments provide a core reference basis for the exploration planning of oil and gas fields, as well as the evaluation of reservoir properties. The wettability determines the micro distribution and original distribution state of fluids in rock pores and also affects the degree of difficulty of fluid injection into reservoir rock pores.

#### 2.4.4. Toxicity Evaluation

The biological toxicity was measured using the DXY-II biological toxicity tester, with the testing method following GB/T 15441-1995 [41] and the grading standard for biological toxicity adhering to Q/SY 111-2007 [42].

## 3. Results and Discussion

### 3.1. Characterization of ASN

#### 3.1.1. Characterization of FT-IR

**Figure 3:**
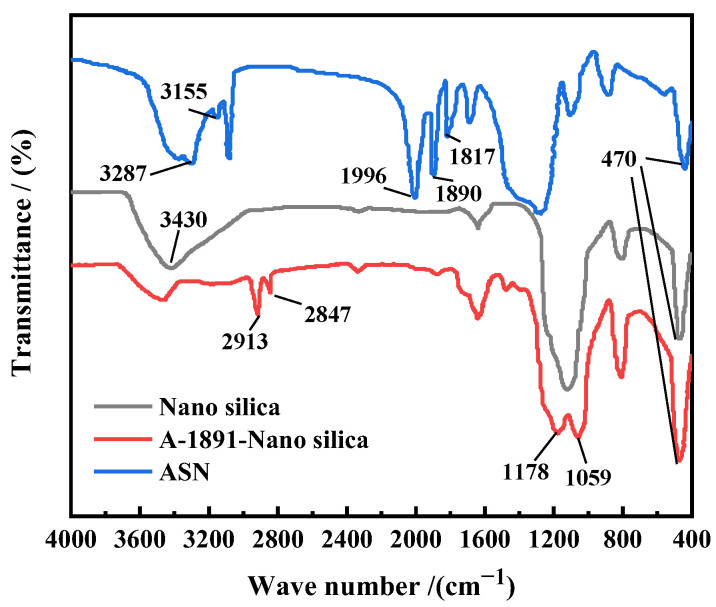
The FT-IR of the sample preparation process.

Figure 3 depicts the infrared spectrum of the nano-SiO_2_ samples before and after surface modification, as well as the nanosealing agent ASN. In the spectra of sample nano silicon and A-1891-nano silicon, the broad peak at 3430 cm^−1^ is a vibrational peak associated with silanol groups and hydrogen bonds. This indicates that there are a large number of OH^-^ groups present on the surface of the nano SiO_2_. After surface modification, the broad peak of 3390–3700 cm^−1^ significantly weakens. After further modification, there are two additional peaks at 2913 and 2847 cm^−1^. These two peaks correspond to the vibrational characteristics of -CH_3_ and -CH_2_ on the modifier. The measurement of 1178 cm^−1^ represents the ester group vibration in A-1891. The specific wavenumbers of 1059 cm^−1^ and 470 cm^−1^ significantly correspond to the vibrational characteristic peaks of the Si-O-Si bond. This indicates that a condensation reaction has occurred between most of the SiO_2_ and the silane-coupling agent, resulting in the successful grafting of the modifier onto the surface of the nano SiO_2_, which has achieved effective surface modification. In the FT-IR of ASN, the peaks at 3287 cm^−1^ and 3155 cm^−1^ represent the characteristic N-H peaks of poly(N-isopropylacrylamide) (PNIPAM), while 1890 cm^−1^ and 1817 cm^−1^ correspond to the distinctive C-O and N-H peaks, respectively. Lastly, the peak at 1996 cm^−1^ signifies the characteristic -COOH peak. Although the sample has been thoroughly extracted with acetone and is free of PNIPAM and AA homopolymers, the FT-IR analysis still indicates their presence. This indicates that the nano SiO_2_ treated with A-1891 surface modification is chemically linked to the PNIPAM-co-AA polymer chains, rather than being a simple physical composite. The copolymerization of A-1891 nano SiO_2_ with PNIPAM and AA under reaction conditions indicates the successful synthesis of the temperature-responsive nanosealing agent ASN.

#### 3.1.2. Particle Size Characterization

**Figure 4:**
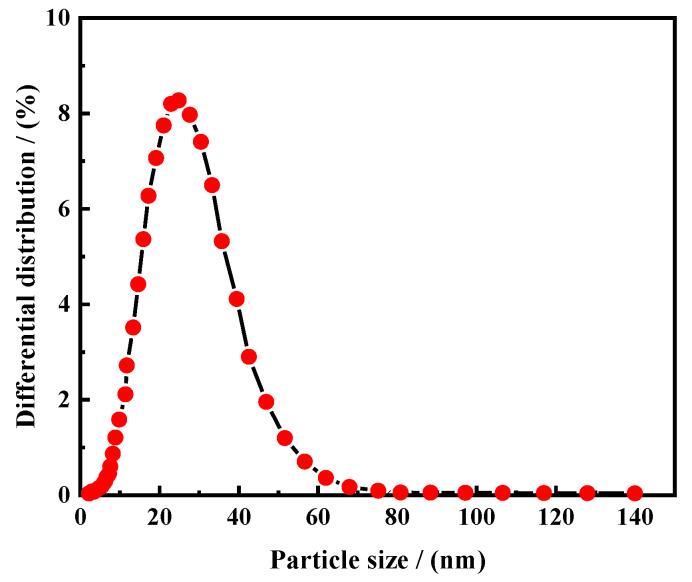
The particle size details of the sample of ASN.

Figure 4 represents the particle size details of the sample of ASN. From the graph, it can be observed that the curve shows a sharp parabolic shape, indicating that the size of the particle diameter is relatively concentrated, and there is no agglomeration phenomenon. In addition, the distribution range of the curve is between 10 and 40 nm. According to the particle size test results, D_50_ = 25 nm, indicating that more than 50% of the synthesized temperature-responsive nanoparticles ASN have a particle size of 25 nm or larger.

#### 3.1.3. Thermogravimetric Analysis (TG)

**Figure 5:**
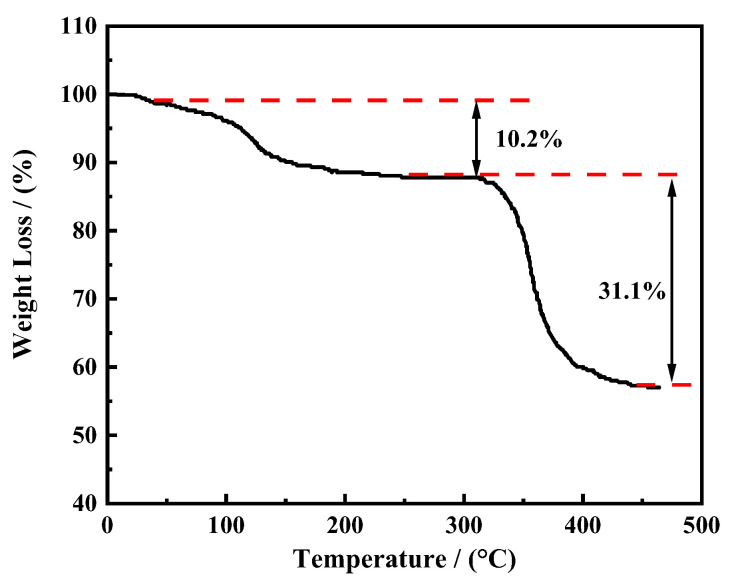
The thermogravimetric analysis of ASN (n_PNIPAM_/n_AA_ = 80/20).

Figure 5 shows the thermogravimetric spectrum of the temperature-responsive nanosealing agent ASN at a PNIPAM/AA molar ratio of 80/20. Upon inspection of the graph, it is evident that there is thermal weight loss near 105 °C, which is related to the adsorption of water and evaporation of solvent. The initial decomposition temperature of the molecular chain of the nanosealing agent is 315.2 °C, indicating good stability. After 441 °C, the weight of the sample basically stabilizes, and only nano SiO_2_ is remaining, with a content of 58.7%. This indicates that ASN itself has good temperature resistance.

#### 3.1.4. ASN Biotoxicity Evaluation

Pursuant to the GB/T 15441-1995 standard, a 1.0% ASN solution was precisely formulated. The results of the biological toxicity test conducted using the DXY-II biological toxicity tester are presented in Table 1. The EC_50_ value, calculated using the standard method with the data in Table 1, is approximately 48,091 mg∙L^−1^. This indicates that the EC_50_ of the 1.0% ASN solution is 48,091 mg·L^−1^. Through reference to the standard Q/SY 111-2007, it was assessed that the 1.0% ASN solution is non-toxic. This indicates that the nanosealing agent ASN is an environmentally friendly treatment agent.

### 3.2. Performance Testing

#### 3.2.1. Sealing Performance Evaluation

(1) Pressure transmission experiment

**Figure 6:**
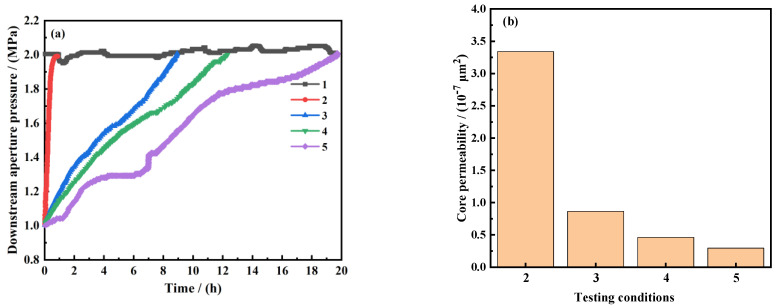
The pressure transfer curve of the shale core in a certain area of Ordos Basin: (**a**) the effect of the sealing agents on the permeability of shale; (**b**) 1 represents the upstream pressure at normal temperature, 2 represents the 4% NaCl at normal temperature, 3 represents the 4% NaCl + 2% commercial nanosealing agents at normal temperature, 4 represents the 4% NaCl + 2%ASN at normal temperature, and 5 represents the 4% NaCl + 2%ASN at 130 °C.

A rock core in the Ordos Basin was selected for pressure transfer experiments to evaluate the dense sealing performance of nanosealing agents. During the pressure transfer process, the downstream test solution used a 4 wt.% NaCI aqueous solution, while the upstream test solution used a base solution of 4 wt.% NaCl aqueous solution or a 4 wt.% NaCl aqueous solution + 2 wt.% ASN nanosealing agent. The sealing capabilities of the temperature-responsive nanosealing agent ASN were rigorously evaluated under both ambient temperature conditions and temperatures exceeding LCST. Figure 6a represents the pressure transmission curve of the test experiment. The shale permeability was calculated using Formula (1). The relevant situation is presented in Figure 6b.

As observed in Figure 6a, the pressure transfer rate of the 4 wt.% NaCl aqueous solution is very fast. After 1 h, the upstream pressure successfully penetrated the rock core. Under room temperature conditions, commercially available nanosealants and ASN can significantly slow down the pressure transfer rate. The time required for upstream pressure to penetrate the rock core has significantly increased, to 8.5 h and 12.5 h. Under the influence of the pressure differential, nanosealing agents penetrate into the pores on the rock surface, forming a physical sealing layer. When the external temperature increases above the lower critical solution temperature, ASN exhibits a more pronounced effect in hindering the pressure transmission rate and reducing the permeability of shale. The time required for upstream pressure to penetrate the rock core increases to 20.3 h. From Figure 6b, it can be seen that the permeability of the affected shale decreases by two orders of magnitude. At this moment, the thermo-responsive molecules of the ASN transition to a hydrophobic state, creating a water-repellent layer on the exterior of the rock. This layer can significantly enhance the wellbore stability, indicating that the nanosealing agent ASN outperforms its commercially available counterparts.

(2) Nuclear magnetic resonance core pore size analysis

**Figure 7:**
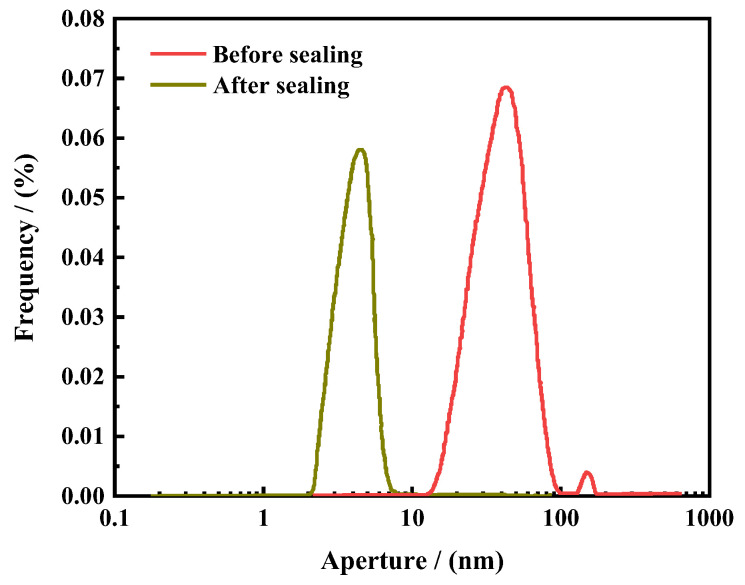
The changes in the NMR pore size distribution after core sealing.

The pore size distribution characteristics before and after shale sealing are shown in Figure 7. According to the nuclear magnetic resonance pore size distribution curve of the core, the pore size distribution range of the shale core before sealing is 30–120 nm, with a median pore size of 70–80 nm. After the action of the nanosealing agent ASN, the aperture situation curve of shale core shifted to the left, with a pore size distribution range of 2–10 nm and a median pore size of around 6 nm. The experiments have shown that ASN can significantly reduce the pore size of shale and make it denser through filling and blocking the nanoscale micropores and microcracks in shale.

#### 3.2.2. Temperature-Sensitive Characteristics

**Figure 8:**
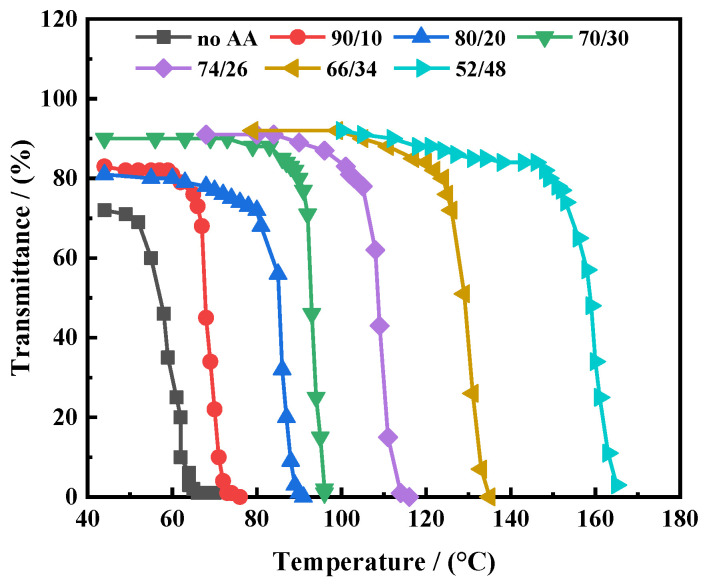
The transmittance temperature relationship curve of the ASN nanosealing agent with different IPAM and AA molar ratios.

Figure 8 depicts the temperature-sensitive characteristics of the nanosealing agent ASN, which exhibits temperature sensitivity due to its varying molar ratios of PNIPAM and AA. As illustrated, the LCST values corresponding to different PNIPAM and AA molar ratios (without AA, 90/10, 80/20, 70/30, 74/26, 66/34, 52/48) of the nanosealing agent ASN are 52 °C, 62 °C, 81 °C, 91 °C, 105 °C, 126 °C, and 153 °C, respectively. Due to the doping of AA, the electrostatic repulsion of the hydrophilic groups in the AA molecular chain increases the temperature at which the molecular chain conformation collapses, resulting in a higher LSCT value. The value indicates that the LCST of ASN progressively rises with an increase in AA content. ASN exhibits a highly sensitive temperature response behavior, displaying a distinct LCST value. The hydrogen bonding and hydrophobic state affect the temperature-sensitive properties of ASN in aqueous solutions [43,44].

#### 3.2.3. Wettability Testing

**Figure 9:**
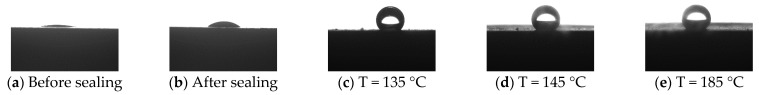
The contact of different conditions.

The wettability test results of the shale-core sealing end face are presented in Table 2 and Figure 9. Deionized water is used to test the contact angle on the surface of the rock core. When the contact angle is greater than 90°, the surface of the rock core is in a hydrophobic state. According to Table 2, The results reveal that, at temperatures below the LCST threshold, the core surface maintains its hydrophilic nature, irrespective of sealing. However, upon reaching temperatures above the LCST, the core surface transitions to a hydrophobic state (contact angle > 90°). This indicates that, when the external temperature surpasses the LCST of the nanosealing agent ASN, the surface changes from hydrophilicity to hydrophobicity and forms a hydrophobic layer. This, in turn, enhances the wellbore stability of the rock strata.

### 3.3. Comprehensive Analysis of the Mechanism of Action

**Figure 10:**
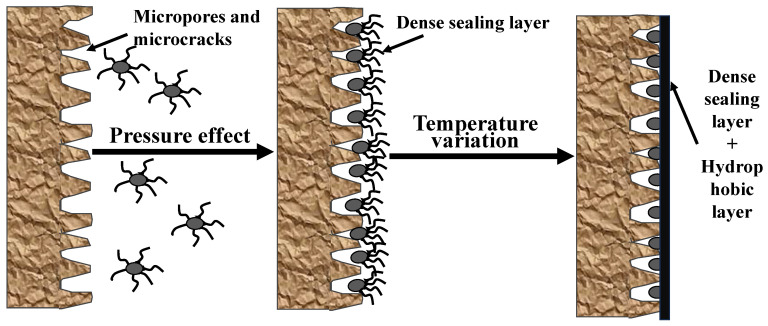
The dual inhibitory mechanism of the ASN blocking agent.

Under the application of a specific pressure, ASN initially penetrates into the diverse pores on the rock surface through irregular deformation, effectively filling and repairing them to form a physical sealing layer. Once the temperature exceeds the LCST of the sealing agent, the ASN surface transitions from hydrophilic to hydrophobic and forms a hydrophobic layer, thereby exerting a chemical hydrophobic effect. Consequently, ASN, under optimal temperature and pressure conditions, is capable of concurrently performing physical sealing and chemical inhibition, resulting in the formation of a continuous and dense pressure-resistant sealing layer around the wellbore. A schematic representation of this process is depicted in Figure 10.

## 4. Conclusions

1. The SiO_2_ nanoparticles were surface-treated with the silane-coupling agent A-1891 to obtain the temperature-responsive nanoparticle ASN, which possesses good thermal stability and strong temperature resistance. ASN is also an environmentally friendly treatment agent.
2. ASN can effectively minimize the pore dimensions of shale and make it denser through entry into the nanoscale micropores and microcracks of shale. When the external temperature surpasses the LCST of the nanosealing agent ASN, its surface undergoes a transition from hydrophilic to hydrophobic and forms a hydrophobic layer. This, in turn, enhances the wellbore stability of the rock strata.
3. Consequently, ASN, under optimal temperature and pressure conditions, is capable of concurrently performing physical sealing and chemical inhibition, resulting in the formation of a continuous and dense pressure-resistant sealing layer around the wellbore.

## Figures and Tables

**Table 1:** The relative luminescence intensity of the 1.0% ASN solution.

Mass Concentration/(10^6^ mg∙L^−1^)	2	3	4	5	6	7	8
Result 1/%	96.84	79.46	61.69	50.55	28.12	24.72	17.30
Result 2/%	90.35	79.18	59.95	41.41	31.61	25.23	12.99
Result 3/%	92.25	82.20	63.21	48.26	31.33	30.22	17.88
Average value/%	93.15	80.28	61.62	46.74	30.35	26.72	16.06

**Table 2:** The wettability testing of rock-core sealing end face.

Test Conditions	Contact Angle (°)
Before sealing	10
After sealing	37
T = 135 °C	136
T = 145 °C	142
T = 185 °C	139

## Data Availability

Data are contained within the article.
